# 肺癌脑转移生存预测因素分析

**DOI:** 10.3779/j.issn.1009-3419.2015.07.08

**Published:** 2015-07-20

**Authors:** 少华 崔, 皓 白, 莉莉 董, 怡卓 赵, 爱琴 顾, 伟 张, 煜清 楼, 丽岩 姜

**Affiliations:** 200030 上海，上海交通大学附属胸科医院呼吸内科 Department of Pulmonary Medicine, Shanghai Chest Hospital, Shanghai Jiao Tong University, Shanghai 200030, China

**Keywords:** 肺肿瘤, 脑转移, 预测因子, 生存期, Lung neoplasms, Brain metastases, Predictor, Survival

## Abstract

**背景与目的:**

脑转移的肺癌患者预后差，采取积极治疗措施后也仅有约6个月的生存时间。本研究对可能影响肺癌脑转移生存的临床因素进行收集和分析，以期为指导临床实践提供一定的研究证据。

**方法:**

回顾性收集上海交通大学附属胸科医院2002年-2008年有病理组织确诊并发生脑转移的肺癌病例。采用*Kaplan-Meier*生存曲线、*Cox*多因素生存分析模型进行生存分析，得到影响脑转移生存的独立预测因素。

**结果:**

年龄、美国东部肿瘤协作组体能状态(Eastern Cooperative Oncology Group Performance Status, ECOG PS)评分、转移间隔时间、转移数目、治疗方法、治疗周期、脑转移症状、颅外转移、脑转移次序能够影响到肺癌脑转移患者的生存。采用*Cox*多因素回归分析得到治疗周期和颅外转移是肺癌脑转移生存时间的独立预测因素。

**结论:**

治疗周期和颅外转移是肺癌脑转移生存时间的独立预测因素。

## Abstract (en)

**Background and objective:**

The prognosis for patients with lung cancer and brain metastases remains poor, with approximately 6 months of survival, despite active measures after treatment. In this study, we determined and analyzed clinical parameters that affect the survival of patients with lung cancer and brain metastases to provide clinical guidance.

**Methods:**

Lung cancer cases with brain metastases were retrospectively collected during 2002 and 2008 from Shanghai Chest Hospital, Shanghai Jiao Tong University. *Kaplan-Meier* method and *Cox* regression were performed for univariate and multivariate analyses, respectively, to explore independent predictors influencing the survival of patients with lung cancer and brain metastases.

**Results:**

Age, Eastern Cooperative Oncology Group performance status (ECOG PS), metastasis interval, number of metastasis, treatment method, treatment period, symptoms of brain metastases, extracranial metastasis, and brain metastasis order were factors that affect the survival of patients with brain metastases as confirmed through the *Kaplan-Meier* method. Treatment periods and extracranial metastasis were independent survival predictors in patients with lung cancer and brain metastasis as indicated by *Cox* proportional hazard model.

**Conclusion:**

Treatment periods and extracranial metastasis were independent predictors of survival of patients with lung cancer and brain metastasis. Treatment periods and extracranial metastasis were independent predictors of survival of patients with lung cancer and brain metastasis.

脑是恶性肿瘤常见的血道转移部位。在确诊时，小细胞肺癌(small cell lung cancer, SCLC)和非小细胞肺癌(non-small cell lung cancer, NSCLC)各约有20%和10%的病例已发生脑转移，而20%的NSCLC病例在病程中会发生脑转移^[1]^。脑转移的肺癌患者预后差，采取积极的治疗措施后也仅有大约6个月的生存时间^[1]^。目前，对单发脑转移主要采用局部放疗或手术方法治疗，而对多发脑转移则采用全脑放疗^[2]^。但有研究^[3-5]^表明，针对单发病灶，多种方法联合治疗的疗效要优于单纯的局部治疗，但联合治疗会造成患者神经功能障碍，且治疗后多数患者会因快速复发而死亡，使脑转移成为肺癌治疗失败的主要原因之一。

影响肺癌脑转移发生和患者生存的因素很多，Robnett等^[6]^对150例接受根治性放化疗的局部晚期NSCLC脑转移风险进行评估，发现临床分期、组织学类型、接受诱导化疗等因素与脑转移发生的风险有关。确诊时临床分期越晚、组织类型为非鳞癌和接受诱导化疗的NSCLC更容易发生脑转移。Ceresoli等^[7]^评估局部晚期接受多学科治疗后NSCLC发生脑转移的风险，结果显示，大多数患者治疗后2年内出现脑转移病灶，转移病灶一般为单发，年龄 < 60岁者脑转移发生的风险更高。Gehan等^[8]^研究表明，年龄、脑转移症状、治疗方法等可能影响到脑转移患者的生存时间。此外，有关肺癌脑转移预后及生存影响因素的研究也有报道^[9, 10]^。

本研究对可能影响肺癌脑转移生存的临床因素进行收集和分析，通过单因素和多因素生存分析，得到影响脑转移生存的独立预测因素，以期为指导临床实践提供一定的研究证据。

## 1 资料与方法

### 1.1 临床资料

回顾性收集上海交通大学附属胸科医院2002年-2008年有病理组织确诊并发生脑转移的肺癌病例。入选标准：经病理组织学确诊的肺癌，组织学类型不限；经头颅增强磁共振成像(magnetic resonance imaging, MRI)或头颅增强计算机断层扫描(computed tomography, CT)、症状、体征或手术证实有脑转移。排除标准：不能确诊肺癌脑转移的病例。收集相关的临床信息，包括性别、年龄、组织类型、分化程度、脑转移部位、原发癌临床分期、美国东部肿瘤协作组体能状态(Eastern Cooperative Oncology Group Performance Status, ECOG PS)评分、脑转移症状、脑转移数目、颅外转移、治疗方法、治疗周期、确诊肺癌到发生脑转移的时间(转移间隔时间)、脑转移次序等。符合条件的病例共323例，其中2例失访，没有纳入最终的生存分析。临床基本资料的情况进行统计详见表 1。其中 > 60岁(67.5%)、男性(67.5%)、腺癌(63.7%)居多；分化程度主要是中分化和低分化；转移范围的情况较均衡，中央型和周围型分别为156例(48.4%)和166例(51.6%)；肺癌确诊时的临床分期集中在Ⅳ期(52.8%)；ECOG PS以0分-2分居多(84.6%)；从确诊肺癌到脑转移发生的间隔时 < 6个月、6个-12个月、 > 12个月的人数分别是62例(32.8%)、76例(40.2%)、51例(27.0%)，其他134例患者确诊时即已发生了脑转移；脑转移发生的部位主要是大脑(92.2%)；转移数目可为单个(165例，51.2%)或多个(157例，48.8%)；治疗方法以化疗和放疗联合(54.2%)多见；治疗≤3周期和≥4周期的患者分别为120例(43.6%)和155例(56.4%)；有脑转移症状出现的有178例(55.3%)；发生颅外转移的有183例(56.8%)；发生脑转移同时也发生其他颅外转移的有103例(57.5%)。

**1:** 脑转移患者的基本临床资料 Basic clinical data of patients with brain metastases

Parameters		*n*	Proportion (%)
Age (yr)	＜60	105	32.5
	≥60	218	67.5
Gender	Male	218	67.5
	Female	105	32.5
Histological type	Adenocarcinoma	206	63.7
	Squamous cell	31	9.6
	Adenosquamous	42	13.0
	Small-cell	23	7.1
	Other	21	6.6
Differentiation	High	8	7.7
	Moderate	56	53.8
	Low	40	38.5
Metastatic range	Central	156	48.4
	Peripheral	166	51.6
Clinical stage	Ⅰ or Ⅱ	30	9.3
	Ⅲa	53	16.5
	Ⅲb	69	21.4
	Ⅳ	170	52.8
ECOG PS	0-1	161	49.9
	2	112	34.7
	3-4	50	15.4
Metastatic interval	< 6 months	62	32.8
	6-12 months	76	40.2
	> 12 months	51	27
Metastatic sites	Cerebrum	296	92.2
	Cerebellum	14	4.4
	Other	11	3.4
Metastatic numbers	Single	165	51.2
	Multiple	157	48.8
Treatment methods	Chemotherapy or Radiotherapy	44	13.6
	Chemotherapy and Radiotherapy	175	54.2
	Surgery	79	24.5
	Palliative Care	25	7.7
Treatment cycles	≤3 cycles	120	43.6
	≥4 cycles	155	56.4
Symptoms of brain metastases	Yes No	178 144	55.3 44.7
Extracranial metastasis	Yes	183	56.8
	No	139	43.2
Brain metastases order	First site	21	11.7
	No first site	55	30.7
	Same time	103	57.6
This study was a retrospective study that can not completely guarantee that all data is complete. Except for some main demographic and pathology information, incomplete data might inevitable exist in the collected characteristic. ECOG PS: Eastern Cooperative Oncology Group performance status.			

### 1.2 随访资料

以发生脑转移的时间为起点，患者死亡的时间为主要研究终点，确立总生存期(overall survival, OS)。在随访截止日期没有死亡的病例，作为删失数据处理。随访方式为门诊随访或电话随访，随访截止日期是2014年5月。

### 1.3 统计分析

采用SPSS 13.0软件进行统计分析。采用*Kaplan-Meier*(K-M)法对每个临床因素(变量)的不同水平进行生存分析，*Log-rank*法进行生存曲线的比较。对单因素生存分析有意义的变量，进入*Cox*比例风险模型进行后续多因素分析，筛选出对脑转移生存具有独立预测作用的因素。本研究所有的检验均采用双侧检验，*P* < 0.05为差异有统计学意义。

## 2 结果

### 2.1 单因素生存分析

截止随访结束，共有307例(95.6%)患者死亡，14例患者存活。中位总生存期为7.8个月，生存曲线见图 1。K-M法对每个变量的不同水平分析和比较(*Log-rank*检验)显示年龄、ECOG PS、转移间隔时间、转移数目、治疗方法、治疗周期、脑转移症状、颅外转移、脑转移次序能够影响肺癌脑转移患者的生存，*P*分别为0.014、 < 0.001、0.017、0.001、 < 0.001、 < 0.001、0.026、0.004、0.001。而性别、病理组织学类型、分化程度、临床分期、转移范围、脑转移部位不能影响脑转移患者的生存，*P*分别为0.627、0.814、0.195、0.052、0.111、0.489(表 2)。在进行*Cox*回归分析时，考虑到临床分期*P*接近0.05，且该因素具有较大的临床意义，因此适当放宽标准，将这一因素也纳入后续*Cox*回归分析。

**1:**
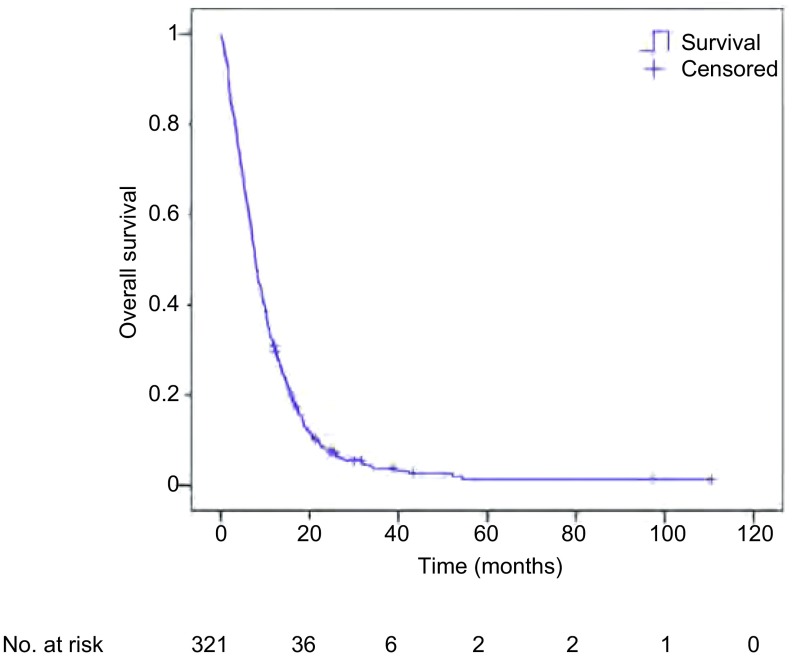
*Kaplan-Meier*法绘制的321例患者的生存曲线 Survival curves by *Kaplan-Meier* method for 321 patients

**2:** *Kaplan-Meier*法单因素生存分析 Univariate survival analysis by *Kaplan-Meier* method

Parameters	Levels	Overall survival (months) (95%CI)	Chi-Square	*P*
Age	≤60 *vs* > 60	6.8 (5.3-8.3) *vs* 8.2 (7.1-9.3)	6.068	0.014
Gender	Male *vs* Female	8.0 (6.9-9.1) *vs* 7.1 (5.6-8.6)	0.236	0.627
Histological type	Adenocarcinoma *vs* Squamous cell *vs* Adenosquamous *vs* Small-cell *vs* Other	7.7 (6.7-8.7) *vs* 8.0 (3.1-12.9) *vs* 7.9 (4.5-11.3) *vs* 8.3 (6.9-9.7) *vs* 6.4 (1.7-11.1)	1.571	0.814
Differentiation	High *vs* Moderate *vs* Low	18.4 (5.6-31.2) *vs* 10.4 (9.1-11.7) *vs* 6.9 (5.2-8.6)	3.266	0.195
Clinical stage	Ⅰ *vs* Ⅱa *vs* Ⅱb *vs* Ⅲa *vs* Ⅲb *vs* Ⅳ	9.5 (3.2-15.8) *vs* 7.6 (6.3-8.9) *vs* 7.9 (3.7-12.1) *vs* 10.1 (7.2-13.0) *vs* 6.1 (4.8-7.4) *vs* 7.4 (6.6-8.2)	10.965	0.052
Metastatic range	Central *vs* Peripheral	7.6 (6.5-8.7) *vs* 7.8 (6.4-9.2)	2.536	0.111
ECOG PS	0 *vs* 1 *vs* 2 *vs* 3 *vs* 4	16.1 (13.7-18.5) *vs* 11.1 (9.8-12.4) *vs* 5.2 (4.4-6.0) *vs* 1.9 (1.5-2.3) *vs* 0.1	432.761	< 0.001
Metastatic interval	≤6 months *vs* 6-12 months *vs* ≥12 months	6.9 (4.7-9.1) *vs* 5.7 (4.6-6.8) *vs* 10.3 (7.8-12.8)	8.190	0.017
Metastatic sites	Cerebrum *vs* Cerebellum *vs* Other	7.7 (6.9-8.5) *vs* 9.4 (7.6-11.2) *vs* 5.2 (0-10.4)	1.432	0.489
Metastatic numbers	Single *vs* Multiple	9.3 (7.7-10.9) *vs* 6.8 (5.7-7.9)	10.975	0.001
Treatment methods	C or R *vs* C and R *vs* S *vs* P	3.0 (2.0-4.0) *vs* 9.0 (7.6-10.4) *vs* 11.0 (9.7-12.3) *vs* 1.7 (1.4-2.0)	194.499	< 0.001
Treatment cycles	≤3 cycles *vs* ≥4 cycles	5.1 (4.6-5.6) *vs* 13.3 (11.8-14.8)	148.612	< 0.001
Symptoms of brain metastases	Yes *vs* No	6.8 (5.4-8.2) *vs* 9.3 (7.6-11.0)	4.939	0.026
Extracranial metastasis	Yes *vs* No	7.1 (6.3-7.9) *vs* 10.1 (8.6-11.6)	8.459	0.004
Brain metastases order	First site *vs* No first site *vs* Same time	13.3 (8.3-18.3) *vs* 5.2 (3.5-6.9) *vs* 6.9 (6.2-7.6)	14.443	0.001
C: chemotherapy, R: radiotherapy, S: surgery, P: palliative care.				

### 2.2 *Cox*多因素回归分析

对包括临床分期在内的10个变量进行*Cox*比例风险模型分析(Enter法)，结果显示，治疗周期和颅外转移是肺癌脑转移生存时间的独立预测因素(*P*均 < 0.001)。而其他因素，包括显示年龄、ECOG PS、转移间隔时间、转移数目、治疗方法、脑转移症状、脑转移次序、临床分期均不是肺癌脑转移生存时间的独立预测因素，P分别为0.541、0.397、0.885、0.287、0.815、0.327、0.361、0.486(表 3)。对*Cox*回归分析得到的两个独立预测因素，治疗周期和颅外转移的K-M单因素分析及*Log-rank*比较结果分别列于图 2和图 3。

**2:**
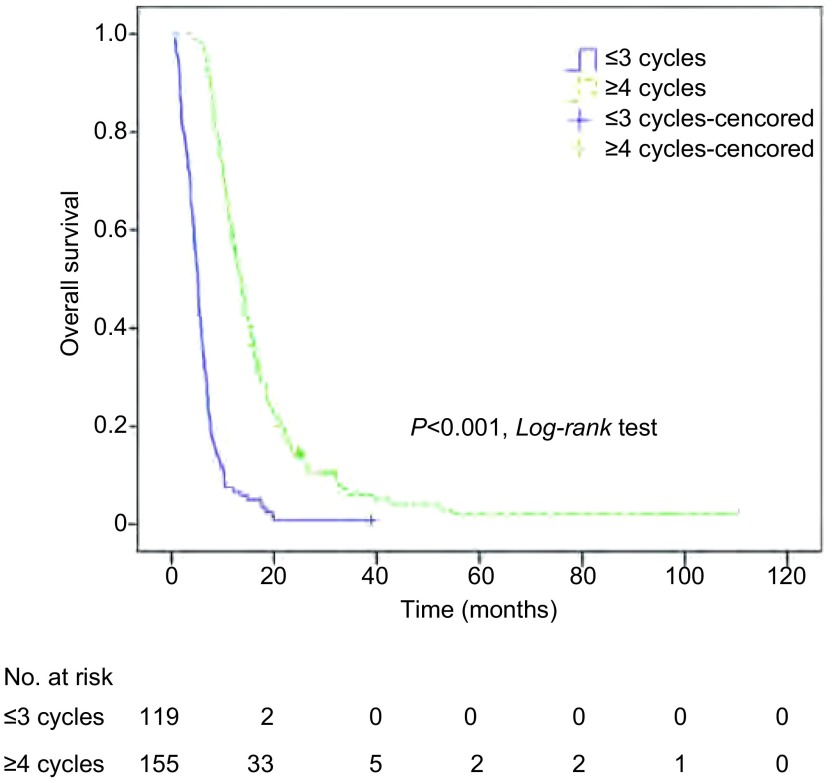
不同治疗周期总生存曲线及比较 *Kaplan-Meier* curves of overall survival for different treatment cycles

**3:**
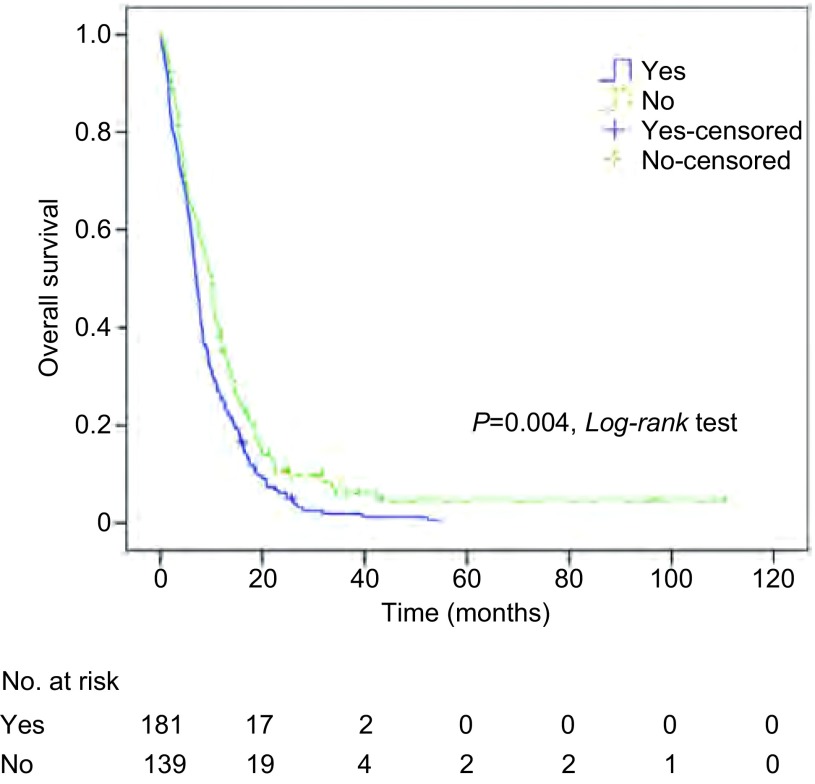
颅外转移因素的总生存曲线及比较 *Kaplan-Meier* curves of overall survival for extracranial metastasis

**3:** *Cox*回归分析结果 Result of *Cox* regression analysis

Parameters	B	SE	Wald	*P*	Exp(B)	95%CI for Exp(B)	
						Lower	Upper
Age	-0.185	0.303	0.374	0.541	0.831	0.459	1.504
Clinical stage	0.072	0.104	0.485	0.486	1.075	0.877	1.318
ECOG PS	0.154	0.182	0.717	0.397	1.167	0.816	1.667
Metastatic interval	-0.025	0.172	0.021	0.885	0.976	0.697	1.366
Metastatic numbers	0.300	0.282	1.133	0.287	1.350	0.777	2.346
Treatment methods	-0.077	0.329	0.055	0.815	0.926	0.486	1.766
Treatment cycles	-2.212	0.351	39.753	< 0.001	0.109	0.055	0.218
Symptoms of brain metastases	0.284	0.290	0.961	0.327	1.329	0.753	2.344
Extracranial metastasis	-0.515	0.182	7.979	< 0.001	0.597	0.418	0.854
Brain metastases order	-0.193	0.212	0.833	0.361	0.824	0.544	1.249

## 3 讨论

脑转移是肺癌治疗失败的主要原因之一^[11-14]^，多种因素可能影响到肺癌脑转移患者的生存。本研究表明，年龄、ECOG PS、转移间隔时间、转移数目、治疗方法、治疗周期、脑转移症状、颅外转移、脑转移次序能够影响到肺癌脑转移患者的生存，而治疗周期和是否有颅外转移是肺癌脑转移生存时间的独立预测因素。

本研究所纳入的病例中腺癌占多数，其原因可能是肺腺癌容易发生血道转移，许多患者常在出现脑转移症状后才发现肺部原发肿瘤。而鳞癌往往先出现淋巴道转移，远处转移发生较腺癌要晚，也不如腺癌多见，所以脑转移患者的多数病理类型是腺癌。分化状态以中低分化为主，因为中、低分化肿瘤的恶性程度高，容易发生脑转移。大多数病例发现肺癌时已属晚期，出现了局部或远处转移，所以Ⅲb期和Ⅳ期病例较多。

K-M生存分析显示年龄和PS评分与肿瘤的预后有关。年轻患者(≤60岁)代谢强，肿瘤发展快，脑内进展的时间相对更短，预后更差。而 > 60岁者肺、脑转移瘤等生长慢，预后反而会好。但以往有报道年龄与脑转移患者的生存无关^[6, 10]^，可能与两组(≤60岁， > 60岁)样本量不均衡有关。PS代表一般机体情况，PS评分低的患者，预后一般较差^[15]^。如晚期肺癌患者，除肺癌本身可能引起的临床表现外，转移的器官也有相应表现，如病理性骨折、胸水等，而且有过手术、肺部慢性疾病的患者肺功能较差，加上肿瘤原因往往卧床不起。而PS相对较好的患者，身体各方面机能都较好，生存期长。从确诊肺癌到脑转移发生的时间和脑转移数目能够间接反映肿瘤的恶性程度，间隔时间越短、转移数目越多，表明肿瘤发展越快，预后也越差。干预措施也会影响脑转移预后，目前已有研究^[16]^结果显示，单纯化疗或放疗的疗效不如化疗联合放疗。本研究结果同样显示，接受单纯放疗或单纯化疗的患者生存期短于化疗联合放疗的患者。表明单一治疗方式对脑转移病灶的控制能力有限，不能延长患者生存，而二者联合治疗是肺癌脑转移患者可选的治疗方法，这可能与放疗能破坏血脑屏障，增加血脑屏障的通透性，从而使化疗药物在脑脊液中达到较高浓度有关，二者联合使用可以起协同作用^[17, 18]^。

本研究中多数患者接受化疗和放疗联合治疗，化疗作为多学科治疗的主要方法，对肺癌脑转移患者的预后存在较大影响。本研究证实治疗≥4个周期的患者的中位总生存时间长于治疗不足4个周期的患者。4个周期内(包括1个周期、2个周期、3个周期)的化疗对肿瘤细胞，包括脑转移细胞的杀伤作用可能不够，容易引起短期进展从而使患者预后不良，生存期缩短。此外，传统的化疗一般是4个-6个周期，认为4个-6个周期的治疗才是比较合适的治疗周期，而过少疗程的治疗对肿瘤控制效果不佳，达不到杀伤或抑制肿瘤细胞生长的目的。此外，考虑到化疗药物能否通过血脑屏障，及肺癌远处转移患者肿瘤的恶性程度可能更高、进展更快，发生脑转移患者治疗的疗程应该更长。因此，治疗超过4个周期的患者预后较好。因此，在化疗毒副反应可以接受的范围内，肺癌脑转移患者采用4个周期的化疗是可取的，能够一定程度延长肺癌脑转移患者的总生存期。

肺癌发生颅外转移常提示预后不良。Tian等^[19]^的研究表明，放疗后肺癌原发灶是否控制，有否肺或骨转移，是影响生存期和预后的因素。此外，近期Bae等^[20]^对2, 832例肺癌术后发生脑转移，但没有颅外转移的NSCLC进行研究，发现腺癌亚型、长的无病生存时间、系统治疗脑转移、局部治疗脑转移(手术或立体定向放射外科)的NSCLC预后好，且它们都是独立预测因素。因此，对仅有脑转移而没有颅外转移患者，应采取积极的治疗措施，延长这部分潜在获益人群的生存。本研究*Cox*多因素分析显示治疗周期和有无颅外转移是肺癌脑转移生存的独立预测因素，其他变量的作用均被掩盖，可能是多个变量间有相互作用的结果，如分化程度可以代表肿瘤的恶性程度，与PS评分、分期等都有一定关系。

Sperduto等^[9]^回顾性分析5, 067例接受脑转移治疗的患者的预后，发现体力状态(卡式评分)、年龄、有无颅外转移和脑转移数目等与肺癌脑转移患者的预后有关。Tang等^[10]^对接受放疗的125例发生脑转移的NSCLC进行研究，结果显示体力状况评分≤2分、癫痫发作、寡转移灶的积极局部治疗(手术或立体定向放射外科治疗)、治疗后续采用全身性治疗的患者预后较好。这些研究得到的结论与本研究有所不同，其原因可能是分析所选入的临床因素不同，导致统计结果的差异。但本研究对临床有一定指导意义，通过K-M单因素生存分析和*Cox*比例风险模型多因素分析，我们发现，治疗周期和颅外转移是肺癌脑转移生存时间的独立预测因素，对存在肺癌脑转移的患者，没有其他部位转移是潜在的治疗获益人群。此外，在平衡疗效和风险的同时，进行4个周期以上的化疗可以延长肺癌脑转移患者的生存。

综上所述，肺癌是一种复杂的疾病，脑转移的发生与肿瘤的异质性密切相关，且多种因素可能影响脑转移患者的预后^[21]^。本研究收集影响肺癌脑转移生存的临床因素，通过单因素和多因素生存分析，得到治疗周期和有无颅外转移是影响脑转移生存的独立预测因素。此外，本研究是回顾性分析，可能掺杂没有考虑到的因素，引起一定的偏倚，还需要今后开展大样本量的前瞻性随机对照临床试验加以证实。
